# Acrokeratosis Paraneoplastica Associated with Cervical Squamous Cell Carcinoma

**DOI:** 10.1155/2016/7137691

**Published:** 2016-12-22

**Authors:** Bryan Squires, Steven D. Daveluy, Michael C. Joiner, Newton Hurst, Michael Bishop, Steven R. Miller

**Affiliations:** Karmanos Cancer Institute, Wayne State University, 4100 John R St., Detroit, MI 48201, USA

## Abstract

*Background*. Acrokeratosis paraneoplastica, or Bazex syndrome, is a paraneoplastic syndrome characterized by cutaneous psoriasiform lesions with associated acral erythema and scale, as well as nail changes, including onycholysis and ungual dystrophy. Its most advanced, severe form involves the trunk, elbows, and knees. It is typically associated with upper aerodigestive tract malignancies in males. Rare cases associated with gynecological cancers have been reported, including uterine adenocarcinoma, as well as ovarian and vulvar squamous cell carcinomas. Cutaneous manifestations often precede cancer diagnosis. In most reported cases, skin changes resolve when the underlying malignancy is adequately treated.* Main Observations*. We present the case of a 56-year-old female diagnosed with acrokeratosis paraneoplastica following the discovery of FIGO stage IIB cervical squamous cell carcinoma (SCC). Scaling, hyperpigmentation, xerosis, and fissuring were noted on the patient's hands, feet, legs, arms, and lower back. Pitting was noted on her fingernails. Her cervical cancer was successfully treated with chemoradiotherapy, after which her cutaneous lesions persisted for two months before resolving.* Conclusions*. The presentation of acrokeratosis paraneoplastica in this context is atypical. Reports of associations with gynecological cancers, as in our patient's case, are exceedingly rare.

## 1. Introduction

Acrokeratosis paraneoplastica, otherwise known as Bazex syndrome, is a paraneoplastic syndrome in which cutaneous lesions appear in association with an underlying malignancy. Early clinical recognition is vital to improving patient outcomes, considering that its manifestations precede cancer diagnosis in 65–70% of affected patients [1–3]. Hyperkeratotic plaques with associated erythema and scale present in a symmetric acral distribution and spread centripetally, accompanied by nail changes, including onycholysis and ungual dystrophy [3–5]. Ears and nails are most commonly affected, followed by the nose, hands, and feet [2]. Skin changes typically improve with treatment of the underlying malignancy. If disease does not improve or recurs, it is important to investigate for recurrence or metastasis [1, 4, 5]. We present the case of a 56-year-old woman with acrokeratosis paraneoplastica associated with cervical squamous cell carcinoma (SCC).

## 2. Case Report

A 56-year-old female presented for evaluation of a scaly, thickened rash on the hands and feet. It started approximately four months previously on the left first finger and rapidly developed on the other hand and both feet. She reported pruritus but no tenderness. Two months into the course of the rash, she was diagnosed with cervical SCC (FIGO stage IIB).

**Figure 1:**
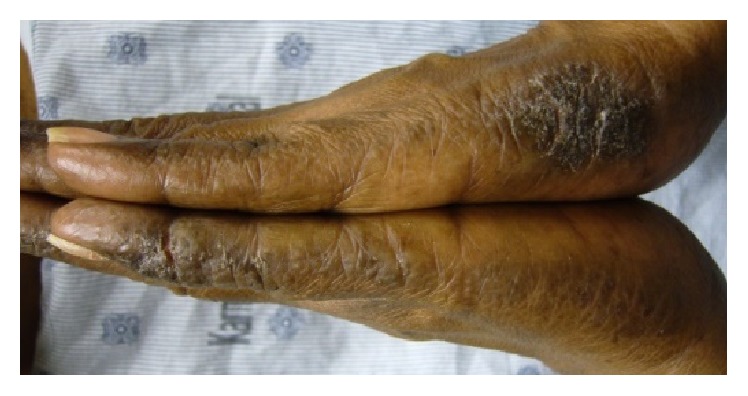
*Acrokeratosis paraneoplastica*. Of note, the patient had scaly hyperpigmented plaques on the ulnar surfaces of the bilateral hands and the periungual skin of the fingers, with some fissuring, as well as irregular pitting of the right fifth fingernails.

**Figure 2:**
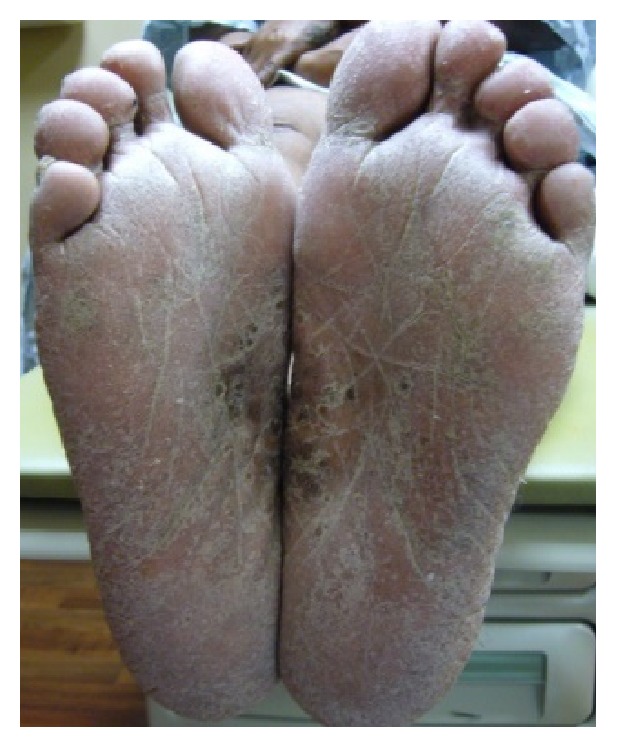
*Acrokeratosis paraneoplastica*. The bilateral plantar feet demonstrated hyperkeratosis extending to the lateral surfaces of the feet and toes.

**Figure 3:**
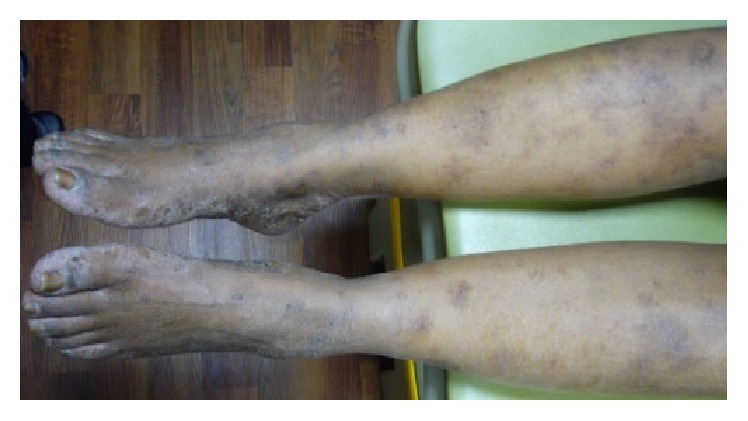
*Acrokeratosis paraneoplastica*. Xerotic scaling and excoriations were present on the bilateral upper arms, lower legs, and lower back. The xerosis was accentuated on the lower legs with fine, polygonal cracks.

Physical exam revealed scaly hyperpigmented plaques on the ulnar surfaces of the bilateral hands and the periungual skin of the fingers, with some fissuring. There was irregular pitting of the right fifth fingernails (Figure 1). The bilateral plantar feet demonstrated hyperkeratosis extending to the lateral surfaces of the feet and toes (Figure 2). Xerotic scaling and excoriations were present on the bilateral upper arms, lower legs, and lower back. The xerosis was accentuated on the lower legs with fine, polygonal cracks (Figure 3).

She was started on betamethasone dipropionate cream for her palms and soles, as well as triamcinolone 0.1% cream for pruritic lesions on the body. Additionally, generous emollients were recommended.

Pelvic external beam radiotherapy (EBRT), brachytherapy, and chemotherapy were initiated to treat her cervical SCC. EBRT was prescribed to 4500 cGy in 180 cGy fractions, followed by five high dose rate (HDR) brachytherapy treatments of 550 cGy to a modified point A of 1.9 cm completed in less than eight weeks, along with six cycles of cisplatin chemotherapy at 40 mg/m^2^.

Her cutaneous manifestations mostly resolved throughout treatment; however, two weeks following completion, lesions were noted to have recurred on her bilateral hands and right foot. Along with the addition of urea cream 40% to her treatment regimen, recommendations were made to continue aggressive moisturization and the application of topical betamethasone dipropionate. Due to continued flaring of her disease, PET scan was performed to rule out persistence or recurrence of her cervical cancer. The PET scan was negative, indicating that her lesions persisted despite remission of her malignancy. Subsequently, her skin responded to therapy and completely resolved approximately two months after completion of chemoradiotherapy.

## 3. Discussion

Acrokeratosis paraneoplastica involves bilateral psoriasiform cutaneous lesions that initially present on acral surfaces, especially the ears, nose, hands, and feet, spreading centripetally [2, 4, 5]. Three clinical stages exist to describe the sequence of cutaneous lesions in relation to the underlying neoplasia: (1) in a locally asymptomatic malignancy, cutaneous lesions present on the nose, ear helices, fingers, toes, and nails; (2) in a locally symptomatic malignancy, palmoplantar surfaces are affected; (3) in a symptomatic malignancy left untreated, lesions advance to the patient's knees, elbows, and trunk [4, 6].

Histopathologic findings are nonspecific and include hyperkeratosis, focal parakeratosis, moderate acanthosis, and lymphohistiocytic infiltration of the upper dermis [1, 4, 5].

Acrokeratosis paraneoplastica is rare, primarily affecting Caucasian men above the age of 40 [2, 5, 7]. In a 2009 review, only twelve cases out of one hundred and forty-five reported involved women [6]. Approximately 80% of cases involve SCC of the upper aerodigestive tract; one retrospective study attributes 48.6% of cases to oropharyngeal and laryngeal cancers, 17% of cases to lung cancer, and 10% of cases to esophageal cancer [3]. In addition, adenocarcinomas of the prostate, stomach, and colon have been implicated [8]. Other rare instances have involved peripheral T-cell lymphoma, Hodgkin's disease, transitional cell carcinoma of the bladder, bronchial carcinoid tumor, thymoma, liposarcoma, metastatic neuroendocrine tumor, cholangiocarcinoma, ductal carcinoma of the breast, and cutaneous SCC [7–9]. Cases associated with gynecologic malignancies are rare and include uterine adenocarcinoma, as well as ovarian and vulvar squamous cell carcinomas [9].

Currently, the pathophysiology of acrokeratosis paraneoplastica is not well understood. One theory involves an autoimmune response resulting from molecular mimicry of tumor antigens to epidermal cell growth factor receptors. Another possible explanation involves direct interaction between the skin and cytokines secreted by tumor cells, such as transforming growth factor and insulin-like growth factor [1, 2, 9]. Its association with human leukocyte antigens A3 and B8 may imply genetic susceptibility [3].

Treating the underlying cancer provides either partial or complete symptom resolution in 90–95% of affected patients [1, 4, 5]. Nail dystrophy improves at a much slower rate than other lesions and may persist indefinitely despite treatment [3, 10]. The recurrence of cutaneous lesions may be a sign of cancer recurrence or metastatic disease [1, 4, 5]. Unsuccessful attempts have been made to utilize topical treatments, including keratolytics, corticosteroids, and UVB radiation. In some cases of treatment-resistant and unresectable cancers, etretinate has provided partial improvement [1, 6].

## Abbreviations

SCC: Squamous cell carcinoma
FIGO: International Federation of Gynecology and Obstetrics
EBRT: External beam radiotherapy
cGy: Centigray
HDR: High dose rate
PET: Positron emission tomography
UVB: Ultraviolet B.
